# Prognostic factors in left-sided endocarditis: results from the andalusian multicenter cohort

**DOI:** 10.1186/1471-2334-10-17

**Published:** 2010-01-22

**Authors:** Juan Gálvez-Acebal, Jesús Rodríguez-Baño, Francisco J Martínez-Marcos, Jose M Reguera, Antonio Plata, Josefa Ruiz, Manuel Marquez, Jose M Lomas, Javier de la Torre-Lima, Carmen Hidalgo-Tenorio, Arístides de Alarcón

**Affiliations:** 1 Infectious Diseases Section, University Hospital Virgen Macarena, Sevilla. Spain; 2 Infectious Diseases Unit, General Hospital Juan Ramón Jiménez, Huelva. Spain; 3 Infectious Diseases Service, University Hospital Carlos Haya, Málaga. Spain; 4 Infectious Diseases Section, University Hospital Virgen de la Victoria, Málaga. Spain; 5 Internal Medicine Service, Hospital Costa del Sol, Marbella. Spain; 6 Infectious Diseases Section, University Hospital Virgen de las Nieves, Granada. Spain; 7 Infectious Diseases Service, University Hospital Virgen del Rocío, Sevilla. Spain

## Abstract

**Background:**

Despite medical advances, mortality in infective endocarditis (IE) is still very high. Previous studies on prognosis in IE have observed conflicting results. The aim of this study was to identify predictors of in-hospital mortality in a large multicenter cohort of left-sided IE.

**Methods:**

An observational multicenter study was conducted from January 1984 to December 2006 in seven hospitals in Andalusia, Spain. Seven hundred and five left-side IE patients were included. The main outcome measure was in-hospital mortality. Several prognostic factors were analysed by univariate tests and then by multilogistic regression model.

**Results:**

The overall mortality was 29.5% (25.5% from 1984 to 1995 and 31.9% from 1996 to 2006; Odds Ratio 1.25; 95% Confidence Interval: 0.97-1.60; p = 0.07). In univariate analysis, age, comorbidity, especially chronic liver disease, prosthetic valve, virulent microorganism such as *Staphylococcus aureus*, *Streptococcus agalactiae *and fungi, and complications (septic shock, severe heart failure, renal insufficiency, neurologic manifestations and perivalvular extension) were related with higher mortality. Independent factors for mortality in multivariate analysis were: Charlson comorbidity score (OR: 1.2; 95% CI: 1.1-1.3), prosthetic endocarditis (OR: 1.9; CI: 1.2-3.1), *Staphylococcus aureus *aetiology (OR: 2.1; CI: 1.3-3.5), severe heart failure (OR: 5.4; CI: 3.3-8.8), neurologic manifestations (OR: 1.9; CI: 1.2-2.9), septic shock (OR: 4.2; CI: 2.3-7.7), perivalvular extension (OR: 2.4; CI: 1.3-4.5) and acute renal failure (OR: 1.69; CI: 1.0-2.6). Conversely, *Streptococcus viridans *group etiology (OR: 0.4; CI: 0.2-0.7) and surgical treatment (OR: 0.5; CI: 0.3-0.8) were protective factors.

**Conclusions:**

Several characteristics of left-sided endocarditis enable selection of a patient group at higher risk of mortality. This group may benefit from more specialised attention in referral centers and should help to identify those patients who might benefit from more aggressive diagnostic and/or therapeutic procedures.

## Background

The diagnostic and therapeutic advances of recent years have only marginally reduced mortality associated with infective endocarditis (IE). Thus, a 30% mortality rate was reported after the introduction of antimicrobial therapy [1] but 50 years later, despite the introduction of new antimicrobial agents and advances in surgical therapy, and even though some recent publications found mortality rates lower than 20%[2,3], it remains around 20-40% in most series [4,5].

During the past few years, several published epidemiological studies have identified a number of prognostic factors related to higher mortality, such as advanced age [6,7], female gender [6], prosthetic valve endocarditis [6,7], *Staphylococcus aureus *aetiology [6-10], comorbidity [6,9], analytical data (leucocytosis [11], hypoalbuminemia [11], C-reactive protein values [12] and elevated ERS [7]), and the development of various complications (heart failure [6-9,12,13], cerebral embolism [7,10,12] renal insufficiency [6], septic shock [10] and paravalvular extension [7]). However, studies investigating prognostic factors for IE frequently have methodological flaws since they are based on short retrospective series, the experience of a single hospital, or use non-uniform diagnostic criteria. Moreover, many of these studies are performed in referral centers, where the most complicated cases are usually treated [14]. More accurate information may be obtained from prospective multicenter studies, in which it is possible to include a large number of cases based on strict diagnostic criteria. Knowledge of potentially modifiable risk factors should help to identify those patients who might benefit from more aggressive diagnostic and/or therapeutic procedures [15]. Our objective was to investigate factors associated with a worse prognosis in a multicenter cohort of left-sided IE patients.

## Methods

### Study design and patients

All patients diagnosed of IE in seven hospitals in Andalusia (South of Spain) were consecutively registered in a uniform database from January 1984 throughout December 2006. Five are tertiary referral hospitals for cardiac surgery and 2 are community hospitals; the latter transferred patients evaluated as being at higher risk for mortality to the referral centres. All cases of left-sided IE defined according to Duke criteria [16] with later modifications [17] for definite and possible IE were included. Patients registered before 1994 were retrospectively evaluated for diagnostic criteria. In the case of relapses, only the first episode was included. Cases with insufficient follow-up (not longer than one month) were excluded.

### Variables and definitions

Data were recorded prospectively by members of the Infectious Diseases services or units in the participating centres using a structured questionnaire and introduced into a common database. The cases were detected from routine review of microbiology, echocardiography and surgery reports. The primary outcome measure was overall mortality during hospitalisation (in-hospital death). All cases with no signs of infection and a negative post-antimicrobial treatment culture were considered cured. Relapses, defined as an IE episode occurring within 6 months of a previous one and caused by the same microorganism, were also recorded. Recurrence was defined as a new IE episode in the same patient and related to a different microorganism. The following independent variables were recorded: age, sex, health care-associated acquisition, type and severity of underlying comorbidity evaluated according to the age-adjusted Charlson index [18], duration of symptoms before diagnosis, septic shock, heart failure, embolism, neurologic manifestations, acute renal failure, echocardiographic findings (vegetations and size, abscess, prosthetic dehiscence and valvular dysfunction), microbiologic data (microorganisms isolated from blood cultures and/or valve tissues, and serologic tests), and treatment (medical and surgical). For analysis purposes, the study period was arbitrary divided in two: 1984 - 1995 and 1996 - 2006.

The additive euroSCORE index [19] were calculated for operative risk evaluation in every patient. Predisposing valvulopathy was defined as any previous valvular heart lesion that predispose to IE. Prosthetic endocarditis was considered as early if it occurred within one year of valve implantation and late otherwise. Heart failure was defined according to Framingham's criteria [20] and was evaluated at admission and during evolution. Septic shock was defined according to standard criteria [21]. Perivalvular extension was defined as the presence of an abscess and/or fistula and/or pseudoaneurism diagnosed by echocardiogram, in surgery or at autopsy. A major symptomatic embolism was considered if the central nervous system, spleen or extremities were affected. Neurologic manifestations were considered only in patients with clinical manifestations related to encephalopathy, meningitis, cerebral infarct or a haemorrhage, and demonstrated by cerebrospinal fluid analysis, radiological test or at autopsy. Severe renal insufficiency was defined as a serum creatinin level >2.5 mg/dl. Left-sided IE were considered nosocomially acquired using previously published criteria [22]. After 1994 transesophagic echocardiography (TEE) was performed in cases with a high clinical probability of IE when transthoracic echochardiography (TTE) was not diagnostic, and routinely in prosthetic IE or when cardiac complications were suspected.

This study was approved by ethical and research committees of all participating hospitals.

### Statistical analysis

The association of the different variables with the outcome was firstly analysed by univariate analysis. Continuous variables were expressed like mean ± standard deviation or like median and interquartile rank in case of abnormal distribution and were analysed using the Student's T test or the Mann-Whitney U test as appropriate. Categorical variables were expressed like absolute numbers and proportion of cases and were compared using the chi-squared or Fisher's test when appropriate. Relative risks with 95% confidence interval for mortality were calculated. Then, multivariate analysis was performed using logistic regression. The multivariate model was built including all variables with a significant association in univariate analysis (p < 0.05) and those considered potentially clinically relevant; modification effects between variables were also studied. The selection of variables was performed using a stepwise backward procedure. All statistical studies were performed using SPSS software (v12.0, Chicago, IL, USA).

## Results

### General features of the cohort

Seven hundred and five case-patients of left-sided IE were included in the study. Only other 8 patients were excluded because insufficient evolution data. Among included cases, 624 (88%) were considered as definite and 81 (12%) as possible IE. Mean (± SD) post-hospitalisation follow-up was 15.75 ± 25.05 months (range: 1-192). The median age of the patients was 56 years (interquartile rank from 41 to 68 years), only 5 patient were ≤ 15 years old. Four hundred and eighty six (69%) patients were male. Native valves were affected in 534 (76%) cases. Of 171 patients (24%) with prosthetic IE, 69 were considered early and 102 late. The aortic valve was affected in 320 (45%) cases, the mitral valve in 299 (42%), and both valves were affected in 86 (11%) patients. Globally, 488 (69%) had a predisposing valvulopathy for IE and 57 (8%) had suffered at least one previous episode of IE. The mean (± SD) age-adjusted Charlson index was 1.84 ± 2.27 (range: 0-10), and the most frequent underlying diseases were: diabetes mellitus (35 patients, 5%), non-valvular heart diseases (32 patients, 5%), chronic liver diseases (32 patients 5%); neoplasia (28 patients, 4%); chronic renal insufficiency (24 patients, 3%); and chronic lung disease (16 patients, 2%). Forty-six patients (7%) were intravenous drug abusers.

Aetiology was established by isolating the causative microorganism from blood culture in 598 (85%) patients, a surgically-explanted valve culture in 70 (10%) and by serologic test in 35 (5%). *Streptococcus viridans *group was the most frequent microorganism isolated, followed by *Staphylococcus aureus*, coagulase-negativestaphylococci and *Enterococcus spp*. (table 1). In 71 cases (10%), aetiology was unknown, because all microbiological studies were negatives.

**Table 1:** Microbiologic aetiology by IE type in 705 patients with left-sided infective endocarditis.

Microorganism	Native	Early Prosthetic	Late Prosthetic	Total
*Streptococci*				

*Streptococcus viridans *group	132 (25)	3 (4)	21 (21)	156 (22)

*Enterococcus spp*	60 (11)	6 (9)	12 (12)	78 (11)

*Streptococcus bovis*	18 (3)	0	1 (1)	19 (3)

*Streptococcus agalactiae*	12 (2)	0	3 (3)	15 (2)

*Others*	21 (4)	0	3 (3)	24 (3)

*Staphylococci*				

*Staphylococcus aureus*	106 (21)	11 (16)	12 (14)	137 (19)

Coagulase-negative *Staphylococci*	45 (8)	34 (50)	23 (23)	102 (15)

Gramnegative bacilli	14 (3)	1 (2)	1 (1)	16 (2)

HACEK Group	5 (1)	0	4 (4)	9 (1)

*Brucella spp*	12 (2)	0	0	12 (2)

*Coxiella burnetii*	12 (2)	0	6 (6)	18 (3)

Fungi	5 (1)	3 (4)	1 (1)	9 (1)

*Other*	12 (2)	4 (6)	4 (4)	20 (3)

Polymicrobial	19 (4)	0	0	19 (3)

Unknown	53 (10)	7 (10)	11 (11)	71 (10)

Total	534 (100)	69 (100)	102 (100)	705

Data are expressed as number of cases (%).

Echocardiography was performed on 698 patients: TTE alone on 409 (58%), TEE alone on 16 (2%) and both TTE and TEE on 273 patients (39%). In 7 patients, no echocardiographic study was performed because endocarditis was not suspected until surgery or necropsy. Overall, TTE was diagnostic in 448 of 682 patients (66%) in which it was performed, and TEE in 251 of 289 (87%). In 156 patients where a TTE study was not diagnostic, a TEE study obtained a defined diagnosis of IE in 122 (78.2%).

Many patients developed complications: 282 (40%) had cardiac failure, 194 (27%) presented neurologic manifestations, 280 (40%) had systemic embolisms (in 147 cases, the central nervous system was affected), 190 (27%) developed acute renal insufficiency, 86 (12.2%) septic shock, and 87 (13%) perivalvular extension (perivalvular abscess, 70; fistula 15; and pseudoaneurism 2).

Valvular surgery was performed in 269 patients (38%). The most frequent indications for surgery were severe heart failure (in 171 patients, 63.5%), followed by valve dysfunction (37 patients, 13.7%), persistent sepsis (21 patients, 7.8%), and perivalvular extension (8 patients, 2.9%). There were several indications for surgery in 27 patients (10.0%). Fifty and three (7.5%) was not operated because of critical situation and high risk intervention.

### Prognosis

Two hundred and eight patients died during their hospital stay (mortality rate, 29.5%). Relapses occurred in 17 patients (2.4%), and the 480 remaining cases (68%) were considered cured. Principal causes of death was: heart failure in 44 patients (21.5%) sepsis in 28 (13.4%) neurologic complications in 22 (10.5%), and by several of these complications in 71(34.1%).

The variables significantly associated with increased mortality in univariate analysis were age, nosocomial acquisition, severity of underlying disease, chronic liver disease, prosthetic valve endocarditis, early prosthetic valve endocarditis, *S. aureus*, *Streptococcus agalactiae *and fungal aetiology, lesser duration of symptoms before diagnosis, development of septic shock, progressive or refractory heart failure, acute renal insufficiency, the presence of cardiac abscess or fistula, and non performed surgery although indicated because of high associated risk (tables 2 and 3). Conversely, *Streptococcus viridans *group aetiology was associated with lower mortality rates. The final multivariate model is shown in table 4; the p value of the Hosmer-Lemeshow goodness-of-fit test for the final model was 0.514.

**Table 2:** Univariate analysis of in-hospital-mortality: patient characteristics and etiology.

*Variable*		Alive N = 497	Deaths N = 208	RR	95% CI	*p*
Age (years)	Mean ± SD	51.6 ± 17	58.8 ± 16	-	-	<0.001

Gender	Male	345 (71)	141 (29)	1.07	0.76-1.52	0.367
	Female	152 (69.4)	67 (30.6)			

Period	1984-1995	196 (74.5)	67 (25.5)	1.25	0.97-1.60	0.070
	1996-2006	301 (68.1)	141 (31.9)			

Hospital	Tertiary	451 (69.3)	200(30.7)	2.07	1.08-3.96	0.008
	Community	46 (85.2)	8 (14.8)			

Nosocomial	Yes	55 (53.4)	48 (46.6)	1.75	1.37-2.24	<0.001
	No	442 (73.4)	160 (26.6)			

Comorbidity	Yes	259 (64.4)	143 (35.6)	1.65	1.28-2.13	<0.001
	No	238 (78.8)	65 (21.5)			

Charlson's index	Mean ± SD	1.56 ±	2,50 ±			<0.001
		2.15	2.42			

Diabetes mellitus	Yes	24 (68.8)	11 (31.4)	1.06	0.64-1.76	0.464
	No	473 (70.6)	197 (29.4)			

Chronic renal insufficiency	Yes	14 (58.3)	10 (41.7)	1.43	0.88-2.33	0.184
	No	483 (70.9)	198 (29.1)			

Chronic liver	Yes	17 (53.1)	15 (46.9)	1.63	1.10-2.40	0.027
Disease	No	480 (71,3)	193 (28,7)			

Valve involved	Aortic	223 (71.9)	118 (29.3)	1.02	0.81-1.28	0.456
	Mitral	274 (69.3)	90 (29.1)			

>1 valve involved	Yes	49 (64.5)	27 (35.5)	1.23	0.89-1.71	0.223
	No	448 (71,2)	181 (28,8)			

Valve type	Prosthetic	104 (60.8)	67 (39.2)	1.48	1.17-1.87	0.001
	Native	393 (73.6)	141 (26.4)			

Prosthetic type	Early	33 (47.8)	36 (52.2)	1.93	1.48-2.50	<0.001
	Late	464 (73)	172 (27)			

*S. aureus*	Yes	70 (51.1)	67 (48.7)	1.96	1.57-2.46	<0.001
	No	427 (75.2)	141 (24.8)			

*S. viridans *group	Yes	140 (89.7)	16 (10.3)	0.29	0.18-0.47	<0.001
	No	357 (65)	192 (35)			

Coagulase-negative *Staphylococci*	Yes	64 (62.7)	38 (37.3)	1.32	0.99-1.75	0.040
	No	433 (71.8)	170 (28.2)			

*Enterococcus spp*.	Yes	52 (66.7)	26 (33.3)	1.14	0.1-1.60	0.254
	No	445 (71)	182 (29)			

*S. agalactiae*	Yes	7 (46.7)	8 (53.3)	1.84	1.12-2.99	0.044
	No	490 (71)	200 (29)			

Fungal	Yes	2 (22.2)	7 (77.8)	2.69	1.86-3.89	0.004
	No	495 (71.1)	301 (28.9)			

Data are expressed as number of cases (%) except when indicated.

**Table 3:** Univariate analysis of in-hospital-mortality: echocardiographic characteristics, complications and treatment.

Variable		Alive N = 497	Deaths N = 208	RR	95% CI	*P*
Duration of symptoms (days)	Mean ± SD	40.5 ± 84	20.3 ± 27.4			<0.001

Septic shock	Yes	24 (27.9)	62 (72.1)	3.05	2.51-	<0.001
	No	473 (76.4)	146 (23.6)		3.70	

Severe heart failure	Yes	101 (57.1)	76 (42.9)	1.75	1.59-	<0.001
(Admission)	No	397 (75,1)	131 (24,9)		3.25	

Severe heart failure	Yes	150 (53.2)	132 (46.8)	2.60	2.05-	<0.001
(Evolution)	No	347 (82)	76 (18)		3.31	

Renal insufficiency	Yes	99 (52.1)	91 (47.9)	2.15	1.79-	<0.001
	No	397 (77.8)	113 (22.2)		2.69	

Neurologic	Yes	113 (57.9)	82 (42.1)	1.70	1.36-	<0.001
manifestations	No	384 (75.3)	126 (24.7)		2.12	

Perivalvular Extension	Yes	44 (51.2)	42 (48.8)	1.82	1.41-2.34	<0.001
	No	453 (73.2)	166 (26.8)			

Vegetations in echocardiography	Present	362 (68.8)	164 (31.2)	1.29	0.95-	0.075
	Absent	133 (75.4)	44 (24.6)		1.68	

Vegetations > 20 mm*	Yes	35 (63.6)	20 (36.4)	1.40	0.96-	0.070
	No	402 (74)	141 (26)		2.04	

Additive euro SCORE	Mean ± SD	7.86 ± 3.65	12.39 ± 3.07			<0.001

Surgical treatment	Yes	180 (67.2)	88 (32.8)	1.19	0.95-	0.07
	No	317 (72.5)	120 (27.5)		1.50	

Not performed surgery¶	Yes	7 (13.2)	46 (86.8)	19,8	8.80-	<0.001
	No	490 (75.2)	162(24.8)	7	44.89	

Data are expressed as number of cases (%) except when indicated.

* Size of vegetations measured in 598 patients. ¶Surgery indicated but not performed

**Table 4:** Multivariate analysis of prognostic factors of in-hospital mortality.

Variable	β	OR	95% CI	*P*
Charlson comorbidity score	0.217	1.24	1.14-1.35	<0.001

Prosthetic endocarditis	0.688	1.99	1.26-3.14	0.003

*S. aureus *aetiology	0.760	2.13	1.30-3.50	0.002

*S. viridans *group aetiology	-0.88	0.41	0.22-0.76	0.005

Severe heart failure (Evolution)	1.691	5.42	3.31-8.86	<0.001

Neurologic manifestation	0.657	1.93	1.24-2.99	0.001

Septic shock	1.445	4.24	2.33-7.70	<0.001

Perivalvular extension	0.914	2.49	1.37-4.53	0.003

Acute renal insufficiency	0.525	1.69	1.09-2.60	0.017

Surgical treatment	-0.685	0.50	0.30-0.84	0.010

## Discussion

Mortality rate of left-sided IE remains high. Although rates lower than 20% have been reported [7,22,23], they usually range between 20 and 30%[4,5] and are even higher in some subgroups; mortality rates of 50% has been reported in early prosthetic valve IE due to *S. aureus *[24]. Crude mortality rate in our series was 29.5%, an intermediate figure considering the intrinsic features of the patients, the number of complicated cases, and the length of the study period. Considering the evolution of mortality over time, we observed a non-significant upward trend, probably related to an increased percentage of cases caused by more virulent organisms such as *S. aureus*, and to a higher frequency of elderly patients with comorbidities.

Previous studies about the prognosis of IE have often been incomplete in their collection of risk factors, have included heterogeneous subtypes (ie, left-side and right-side endocarditis) or have been carried out on patients treated in a single reference center. Only few multicenter cohort with multivariate analysis studies including large numbers of patients have been reported [6,7,9,10,13,25], of which one included only patients admitted to the ICU [10] and another only patients with prosthetic valve IE [12]. This prospective multicenter study includes cases from both referral and community hospitals. We also include IE in both prosthetic and native valves and we have included most factors found in previous studies, such as patient-related variables, aetiology, and the development of different complications.

A Charlson index of more than 2 points has been reported to be an independent risk factor for mortality in previous studies [6,9]. We used the age-adjusted index on the grounds that age may be related to prognosis [26] and also found it to be related with increased risk of death. Many of our patients had more than one underlying condition. Insulin-dependent diabetes mellitus has been observed to be a risk factor in one study [27] but we did not find such association in ours. On the other hand, a very high mortality rate was found among patients with chronic liver disease (47%), similarly to what has been reported in another Spanish series [28].

Prosthetic valve IE, particularly early episodes and those caused by *S. aureus*, are usually associated with a worse prognosis than native valve IE [23,29]. Crude mortality of prosthetic valve IE in our series was almost 3 times higher than for native IE, corroborating the results of previous studies [12,29,30]. Furthermore, *S. aureus *was independently associated with increased mortality, whilst *Streptoccocus viridans *group was a protective factor. Our results confirm another recent collaborative multicenter study where mortality for IE was higher when due to *S. aureus *(22.4% vs 14.6% for other aetiologies)[30]; association of this microorganism with life-threatening complications, such as septic shock and neurologic manifestations have also been reported [31]. All these data emphasise the prognostic relevance of this microorganism in IE.

Heart failure has been found to be a strong marker for increased mortality in most studies [8]. In contrast to Chu *et al *[7], we did not find heart failure at admission to be associated with a worse prognosis. A possible explanation for this is that heart failure at admission, when due to treatable disorders such as arrhythmia, fluid overload, etc, may be controlled by medical treatment. On the other hand, the development of moderate to severe heart failure during evolution was related to increased mortality. We think that development of heart failure during evolution is probably due in most cases to progressive valve dysfunction, which would only be modifiable by surgery. In fact, surgery was a protective factor in our study. Surgical treatment was a protective factor in only two previous studies [8,9], particularly in complicated cases with severe heart failure [32]. The fact that surgery is an independent protective factor for mortality in our study is possibly related to the high number of complicated cases included with heart failure due to valvular dysfunction. However this date must be interpreted with caution since this is an observational study; in another recent study [8], surgery during episode of IE either was associated with better prognosis when this variable was analysed in a multivariable regression modelling.

The exact impact of surgical treatment in IE is controversial and difficult to assess, since evidence is based in observational studies but not in clinical trials. Some new methods of analysis, such as the use of propensity score, have been applied in some studies in order to improve the control of confounding. However the results of these studies are conflicting probably because of methodological differences, as has been recently reviewed [33].

Neurological complications are frequent and associated with a worse prognosis [7,10,12]. In this study, as found in others, about one quarter of patients developed neurologic complications [34]. We considered this complications only when it was clinically relevant, and not only like alterations in imaging techniques. Embolic phenomena are known to be more frequent at admission and during the first week, to occur despite proper medical treatment, and to be associated with worse prognosis, as was the case in our series.

Septic shock, which was developed by about 10% of our patients, was independently associated with increased mortality. This variable has only been studied previously in a report detailing IE patients admitted to the ICU [10], probably because it is not a frequent complication and cannot be evaluated as an independent risk factor in series with a low number of cases. As septic shock has been associated in general patients with mortality rate of about 50% [35], we consider that this variable helps to identify a high-risk subgroup of patients that may benefit from aggressive management, such as early identification of severe sepsis and triggering evidence-based protocolised care [36].

Vegetations observed in echocardiography have been previously associated to a poor outcome [8] although one study found only large-sized vegetations to be an independent prognostic factor [6]. In our study, a vegetation size of >20 mm almost reached statistical significance in univariate analysis but did not in multivariate analysis; a limitation of our study is that size of vegetations was not measured in all cases.

Perivalvular extension of infection (abscess and/or fistula) has traditionally been seen as an unfavourable prognostic factor [7,37] although less so in more recent studies [25,38]. As in another recent large study [7] we found this complication to be an independent risk factor for mortality, which may be related to a higher post-surgical mortality associated with the intrinsic technically difficulties of these cases. This is a variable that identify another high-risk subgroup of patients, and must be suspected in presence of risk factors for this complications like prosthetic IE and aortic localization [25].

Our study have some limitations: First, changes in the clinical management of IE may have occurred during the long study period, although the inclusion of this variable in the multivariate model did not affect the results. Second, the majority of cases were included from referral centers so that the percentage of complicated cases may have been overestimated. However, other multicenter studies only include cases from reference centers and, even if represented by a small percentage, our series probably better reflects the overall clinical reality. Third, as TEE was not systematically performed, evaluation of echocardiographic data (size of vegetations, perivalvular extension) may be biased. And fourth, it is an observational study, so some results, such as the protective effect of surgery, must be interpreted cautiously.

## Conclusions

We believe that our results may help identify IE patients at increased risk of in-hospital death by using data that are usually bedside available, which identify candidates for a more aggressive management in referral centers, with experience that includes active search for septic and hemodynamic complications and early surgical treatment when indicated.

## Competing interests

The authors declare that they have no competing interests.

## Authors' contributions

JGA: carried out conception and design, analysis, interpretation of data and draft the manuscript. JRB: carried out design, analysis and revising the manuscript. FJMM: carried out analysis and interpretation of data, and revising the manuscript. JMR, AP, JR, JML, JTL, CHT: carried out acquisition of data and revising the manuscript. AA: carried out acquisition, analysis and interpretation of data, and revising it critically.

All authors have given final approval of the version to be published.

## Pre-publication history

The pre-publication history for this paper can be accessed here:

http://www.biomedcentral.com/1471-2334/10/17/prepub

## References

[B1] Durack DT Evaluating and optimizing outcomes of surgery for endocarditis JAMA 2003 290 3207 3208 10.1001/jama.290.24.3250 14693880

[B2] Bouza E Menasalvas A Muñoz P Vasallo FJ Moreno MM García-Fernández MA Infective endocarditis - A prospective study at the end of the twentieth century Medicine (Baltimore) 2001 80 298 307 10.1097/00005792-200109000-00003 11552083

[B3] Hoen B Alla F Selton-Suty C Béguinot I Bouvet A Briaçon S Casalta JP Danchin N Delahaye F Etienne J Le Moing V Leport C Mainardi JL Ruimy R Vandenesh F Changing profile of infective endocarditis. Results of a 1-year survey in France JAMA 2002 288 75 81 10.1001/jama.288.1.75 12090865

[B4] Tornos P Lung B Permayer-Miralda G Barón G Delahaye F Gohlke-Bárwolf Ch Butchart EG Ravaud P Infective endocarditis in Europe: lessons from the Euro Heart Survey Heart 2005 91 571 575 10.1136/hrt.2003.032128 15831635 PMC1768869

[B5] Cabell CH Jollis JG Peterson GE Corey GR Anderson DJ Sexton DJ Woods CW Reller LB Ryan T Fowler V Changing patient characteristics and the effect on mortality in endocarditis Arch Intern Med 2002 162 90 94 10.1001/archinte.162.1.90 11784225

[B6] Thuny F Di Salvo G Belliard O Avierinos JF Pergola V Rosenberg V Casalta JP Gouvertnet J Derumeaux G Iarussi D Ambrosi P Calabro R Riberi A Collart F Metras D Lepidi H Raoult D Harle JR Weiller PJ Cohen A Habib G Risk of embolism and death in infective endocarditis: Prognostic value of echocardiography. A prospective multicenter study Circulation 2005 112 69 75 10.1161/CIRCULATIONAHA.104.493155 15983252

[B7] Murdoch DR Corey GR Hoen B Miró JM Fowler V Bayer A Clinical presentation, etiology, and outcome of infective endocarditis in the 21^st ^century Arch Intern Med 2009 169 463 473 10.1001/archinternmed.2008.603 19273776 PMC3625651

[B8] Chu VH Cabell ChH Benjamin DK Kuniholm EF Fowler V Engemann J Sexton DJ Corey GR Wang A Early predictors of in-hospital deaths in infective endocarditis Circulation 2004 109 1745 1749 10.1161/01.CIR.0000124719.61827.7F 15037538

[B9] Hasbun R Vikram HR Barakat LA Buenconsejo J Quagliarello VJ Complicated left-sided native valve endocarditis in adults. Risk classification for mortality JAMA 2003 289 1933 194 10.1001/jama.289.15.1933 12697795

[B10] Mourviller B Trouillet JL Timsit JF Baudot J Chastre J Regnier B Gilbert C Wolff M Infective endocarditis in the intensive care unit: clinical spectrum and prognostic factors in 228 consecutive patients Intensive Care Med 2004 30 2046 2052 10.1007/s00134-004-2436-9 15372147

[B11] Wallace SM Walton BI Kharbanda RK Hardy R Wilson AP Swanton RH Mortality from infective endocarditis: clinical predictors of outcome Heart 2002 88 53 60 10.1136/heart.88.1.53 12067945 PMC1767155

[B12] Heiro M Helenius H Hurme S Savunem T Engblom E Nikoskelainen J Kotilainen P Short-term and one-year outcome of infective endocarditis in adult patients treated in a Finish teaching hospital during 1980-2004 BMC Infectious Diseases 2007 7 78 10.1186/1471-2334-7-78 17640339 PMC1947990

[B13] Habib G Tribouilloy C Thuny F Brahim A Amazouz M Remadi JP Nadji G Casalta JP Coviaux F Avieiros JF Lescure X Riberi A Weiller PJ Metras D Raoult D Prosthetic valve endocarditis: who needs surgery? A multicentre study of 104 cases Heart 2005 91 954 959 10.1136/hrt.2004.046177 15958370 PMC1769001

[B14] Hayden JA Côte P Bombardier C Effect of the quality of prognosis studies in systematic reviews Ann Intern Med 2006 144 427 437 16549855 10.7326/0003-4819-144-6-200603210-00010

[B15] Granowitz EV Longworth DL Risk Stratification and bedside prognostication in infective endocarditis JAMA 2003 289 1991 1993 10.1001/jama.289.15.1991 12697803

[B16] Durack DT Lukes AS Brigth DK New criteria for diagnosis of infective endocarditis: utilization of specific echocardiographic findings Am J Med 1994 96 200 209 10.1016/0002-9343(94)90143-0 8154507

[B17] Prendergast BD Diagnostic criteria and problems in infective endocarditis Heart 2004 90 611 613 10.1136/hrt.2003.029850 15145855 PMC1768277

[B18] Charlson ME Pompei P Ales KL Makenzie CR A new method of classifying prognosis comorbidity in longitudinal studies: development and validation J Chron Dis 1987 40 378 383 10.1016/0021-9681(87)90171-8 3558716

[B19] Roques F Nashef SAM Michel P Gauducheau E Vicentiis C Baudet E Cortina J David M Fainechney Gabrielle F Gams E Harjula A Jones MT Pintor PP Salamon R Thulin L Risk factors and outcome in European cardiac surgery: analysis of the EuroSCORE multinational database of 19030 patients Eur J Cardiothorac Surg 1999 15 816 823 10.1016/S1010-7940(99)00106-2 10431864

[B20] Ho KK Pinsky JL Kannel WB Levy D The epidemiology of heart failure: the Framingham Study J Am Coll Cardiol 1993 22 4 Suppl A 6A 13A 8376698 10.1016/0735-1097(93)90455-a

[B21] Bone RC Balk RA Cerra FB Dellinger RP Fein AM Knaus WA Schein RM Sibbald WJ American College of Chest Physicians/Society of Critical Care Consensus Conference: definitions for sepsis and organ failure and guidelines for the use of innovative therapies in sepsis Crit Care Med 1992 20 864 874 1597042

[B22] Ben Ami R Giladi M Carmeli Y Orni-Wasserlauf Siegman-Igra Y Hospital-acquired infective endocarditis: should the definition be broadened? Clin Infect Dis 2004 38 843 850 10.1086/381971 14999629

[B23] Anguita M Torres F Castillo JC Delgado M Mesa D Ruiz M Romo E Arizón JM Pronóstico a corto y largo plazo de la endocarditis infecciosa en pacientes no usuarios de drogas por vía parenteral. Resultados durante un período de 15 años (1987-2001) Rev Esp Cardiol 2006 58 1188 1196 10.1157/13079913 16238987

[B24] Chirouze C Cabell CH Fowler VG Khayat N Olaison L Miró JM Habib G Abrutyn E Eykyn S Corey GR Selton-Suty C Hoen B the International Collaboration on Endocarditis Study Group Prognostic factors in 61 cases of *Staphylococcus aureus *prosthetic valve Infective Endocarditis from the International Collaboration on Endocarditis Merged Database Clin Infect Dis 2004 38 1323 1327 10.1086/383035 15127349

[B25] Graupner C Vilacosta I San Román JA Ronderos R Sarriá C Fernández C Mújica R Sanz O Sanmartín JV González Pinto A Periannular extensión of infective endocarditis J Am Coll Cardiol 2002 39 1204 1211 10.1016/S0735-1097(02)01747-3 11923047

[B26] Di Salvo G Thuny F Rosembberg V Pergola V Belliard O Derumeaux G Cohen A Iarussi D Giorgi R Casalta JP Caso P Habib G Endocarditis in the elderly: clinical, echocardiographic, and prognostic features Eur Heart J 2003 24 1576 1583 10.1016/S0195-668X(03)00309-9 12927193

[B27] Kourany W Miro JM Moreno A Corey GR Pappas PA Abrutyn E Hoen B Habib G Fowler VG Sexton DJ Olaison L Cabell ChH the ICE MD Investigator Influence of diabetes mellitus on the clinical manifestations and prognosis of infective endocarditis: A report from the International Collaboration on Endocarditis-Merged Database Scand J Infect Dis 2006 38 613 619 10.1080/00365540600617017 16857604

[B28] Pérez de Isla L Zamorano JL Almería C Rodrigo JL Piedra I Aubele A Endocarditis infecciosa en pacientes con hepatopatía crónica: valoración clínica y pronóstica Rev Esp Cardiol 2003 56 794 800 10.1157/13050334 12892625

[B29] Lalani T Kanafani ZA Chu VH Moore L Corey GR Pappas P Woods CW Cabell CH Hoen B Selton-Suty C Doco-Lecompte T Chirouze C Raoult D Miro JM Mestres CM Olaison L Eykyn S Prosthetic valve endocarditis due to coagulase-negative staphylococci: findings from the International Collaboration on Endocarditis Merged Database Eur J Clin Microbiol Infect Dis 2006 25 365 368 10.1007/s10096-006-0141-z 16767483

[B30] Fowler VF Miró JM Hoen B Cabell CH Abrutyn E Rubistein Corey GR Spelman D Bradley SF Barsie B Pappas PA Anstrom KJ Wray D Fortes CQ Anguera I Athan E Jhones Ph Meer JTM Van der Elliott TS Levine DP Bayer AS for the ICE investigator Staphylococcus aureus endocarditis. A consequence of medical progress JAMA 2005 293 3022 3061 10.1001/jama.293.24.3012 15972563

[B31] Nadji G Remadi JP Coviaux F Mirode AA Brahim A Enriquez-Sarano M Tribouilloy C Comparison of clinical and morphological characteristic of *Staphylococcus aureus *endocarditis with endocarditis caused by other pathogens Heart 2005 91 932 937 10.1136/hrt.2004.042648 15958364 PMC1768988

[B32] Vikram HR Buenconsejo J Hasbun R Quagliarello VJ Impact of valve surgery on 6-month mortality in adults with complicated, left-side native valve endocarditis: a propensity analysis JAMA 2003 290 3207 10.1001/jama.290.24.3207 14693873

[B33] Tleyjeh IM Kashour T Zimmerman V Steckelberg JM Wilson WR Baddour LM The role of valve surgery in infective endocarditis management: A systematic review of observational studies that included propensity score analysis Am Heart J 2008 156 901 909 10.1016/j.ahj.2008.06.031 19061705

[B34] Heiro M Nikoskelaimen J Engblom E Kotilaimen E Marttila R Kottilaimen P Neurologic manifestations of infective endocarditis. *A *17-year experience in a teaching hospital in Finland Arch Intern Med 2000 160 2781 2787 10.1001/archinte.160.18.2781 11025788

[B35] Vincent JL Sakr Y Sprung CL Ranieri M Reinhart K Gerlach H Sepsis in European intensive care units: results of the SOAP study Crit Care Med 2006 34 344 353 10.1097/01.CCM.0000194725.48928.3A 16424713

[B36] Nguyen HB Rivers EP Abrahamian FM Moran GJ Abraham E Trzeciak SH Bryant H David T Huang DT Osborn T Stevens D Talan DA Emergency Department Sepsis Education Program and Strategies to Improve Survival (ED-SEPSIS) Working Group Severe sepsis and septic shock: Review of the literature and emergency department management guidelines Ann Emerg Med 2006 48 28 54 16781920 10.1016/j.annemergmed.2006.02.015

[B37] San Román JA Lopez J Vilacosta I Luaces M Sarriá C Revilla A Ronderos R Stoerman W Gómez I Fernández-Avilés F Prognostic stratification of patients with left-sided endocarditis determined at admission Am J Med 2007 120 e1 369 10.1016/j.amjmed.2006.05.071 17398233

[B38] Choussat R Thomas D Isnard R Michel PL Lung B Hanania G Mathieu P David M du Roy de Chaumaray T De Gevigney G Le Breton H Logeais Y Pierre-Justin E Riberolles C Morvan Y Bischoff N Participants in the Perivalvular Abscesses French Multicentre Study Perivalvular abscesses associated with endocarditis. Clinical features and prognostic factors of overall survival in a series of 233 cases Eur Heart J 1999 20 232 241 10.1053/euhj.1998.1240 10082156

